# Sleep Disorders in Adults with Down Syndrome

**DOI:** 10.3390/jcm10143012

**Published:** 2021-07-06

**Authors:** Sandra Giménez, Miren Altuna, Esther Blessing, Ricardo M. Osorio, Juan Fortea

**Affiliations:** 1 Multidisciplinary Sleep Unit, Respiratory Department, Hospital de la Santa Creu i Sant Pau, 08041 Barcelona, Spain; 2 Sant Pau Memory Unit, Department of Neurology, Hospital de la Santa Creu i Sant Pau, Biomedical Research Institute Sant Pau, Universitat Autònoma de Barcelona, 08193 Barcelona, Spain; maltuna@santpau.cat (M.A.); JFortea@santpau.cat (J.F.); 3 Center of Biomedical Investigation Network for Neurodegenerative Diseases (CIBERNED), 28031 Madrid, Spain; 4 Department of Psychiatry, NYU Langone Health, New York, NY 10016, USA; Esther.Blessing@nyulangone.org (E.B.); Ricardo.Osorio@nyulangone.org (R.M.O.); 5 Barcelona Down Medical Center, Fundació Catalana de Síndrome de Down, 08029 Barcelona, Spain

**Keywords:** Down syndrome, aging, sleep disorders, obstructive sleep apnea, insomnia, Alzheimer’s disease

## Abstract

Sleep disorders, despite being very frequent in adults with Down syndrome (DS), are often overlooked due to a lack of awareness by families and physicians and the absence of specific clinical sleep guidelines. Untreated sleep disorders have a negative impact on physical and mental health, behavior, and cognitive performance. Growing evidence suggests that sleep disruption may also accelerate the progression to symptomatic Alzheimer’s disease (AD) in this population. It is therefore imperative to have a better understanding of the sleep disorders associated with DS in order to treat them, and in doing so, improve cognition and quality of life, and prevent related comorbidities. This paper reviews the current knowledge of the main sleep disorders in adults with DS, including evaluation and management. It highlights the existing gaps in knowledge and discusses future directions to achieve earlier diagnosis and better treatment of sleep disorders most frequently found in this population.

## 1. Introduction

Sleep problems are very frequent in people with Down syndrome (DS). Insomnia, excessive daytime sleepiness, and abnormal movements during sleep are present during their whole lifespan [1,2]. While there is not a specific sleep pathology associated with DS, individuals with DS are more prone to developing certain types of sleep disorders from infancy [3]. The pattern and etiology (both psychological and physical causes) vary with age. In childhood, sleep disruption is more frequent due to behavioral sleep problems, while in adulthood, sleep-related breathing disorders are more frequent [4,5]. As in the general population, in people with DS, adequate identification of the underlying sleep disorder is essential for an appropriate therapeutic approach [2,6]. Most of the sleep studies in DS have been performed on children and were mainly based on parental reports, which have a weak correlation with objective polysomnography results [7]. There are fewer reports on the prevalence, severity, and health consequences of sleep disorders in adults with DS despite the increased life expectancy in this population [8]. Healthcare providers must be alert to the emergence of new sleep problems or the worsening of pre-existing ones in adults with DS [9].

Sleep disorders have a negative impact on general health and mental, behavioral, and cognitive functions, especially in older individuals [10]. They can contribute to decreased autonomy, more social isolation, and family burden [5]. Sleep problems can also have an impact on other comorbidities associated with DS [2]. Most importantly, adults with DS are at ultra-high risk of developing Alzheimer’s disease due to the extra copy of the amyloid precursor protein gene coded in chromosome 21 [11,12]. Growing evidence suggests that sleep disturbances can accelerate the progression to AD dementia, and AD can also aggravate sleep problems in a feed-forward loop [13]. The diagnosis and treatment of sleep disorders in DS are therefore essential to prevent additional consequences and comorbidities. Nevertheless, sleep problems in adults with DS are rarely successfully treated due to a lack of awareness by physicians and caregivers, who often misinterpret sleep problems as normal in aging adults with DS [14].

In this paper, we review the current knowledge of the main sleep disorders in adults with DS and their evaluation and management. We aim to increase awareness of the importance of preserving good sleep quality for healthy aging. Clinical and research gaps are highlighted and recommendations are made to achieve earlier diagnosis and better treatment of sleep disorders in adults with DS.

## 2. Methodology

**Figure 1:**
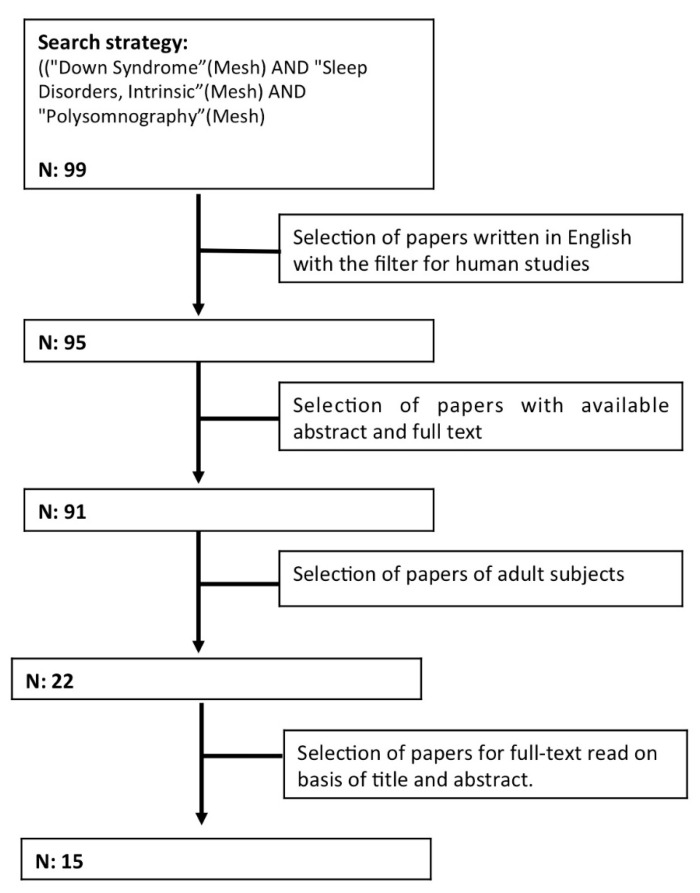
Flow diagram of the literature search.

We performed a literature review using PubMed and Web of Science (WOS) combining search strategies with the following MeSH terms: “Down syndrome”, “sleep disorders, intrinsic”, and “polysomnography” (Figure 1).

We included papers with data on individuals with DS older than 18 years who underwent a sleep study. We did not apply a time restriction, and we included original articles and review articles written in English available online the day of the search (1 May 2021). Only articles on humans were considered. Articles were not considered if the abstracts were not available for screening. After we read the titles and abstracts, papers that met the inclusion criteria were selected for full-text review. Case reports, abstracts, letters to editors, and unpublished studies were excluded. Papers specifically addressing sleep disorders in adults with DS were included in this review. In addition to reading the papers initially selected according to the established search criteria, we also consulted the articles referenced in them. Salient articles are listed in Table 1.

The main goal of this paper was to focus on adults with DS. However, given the limited amount of research on this topic, in the absence of evidence on adults with DS, some works regarding sleep disorders in children with DS and in the general population are also discussed in order to contextualize, to make comparisons, and to provide recommendations.

The review is divided into three parts:

1. Insomnia and behavioral sleep disturbances.
2. Sleep breathing disorders.
3. Sleep movement disorders.

In each section, we first review the available data on the clinical characteristics, then review the screening and diagnostic criteria, and conclude by providing treatment recommendations and indicating relevant gaps in knowledge.

## 3. Sleep Disorders

### 3.1. Insomnia and Behavioral Sleep Disturbances

Insomnia is defined by difficulties with sleep onset and/or sleep maintenance [12].

Behavioral sleep disturbances (BSD) are the most common cause of pediatric insomnia, with bedtime resistance, delayed sleep onset, and nighttime awakening present in 52 to 69% of children with DS [6,7]. In adults with DS, the prevalence of BSD ranges from 13 to 86%, depending on the subject’s age and the definitions and diagnostic measures used in the investigation. With aging, the difficulties associated with maintaining sleep and early morning awakening increase, in part, in relation to age-related comorbidities [25]. Psychological factors, medical problems or pharmacological therapies can increase night awakenings, and a higher prevalence of insomnia and BSD has been reported in older adults [5,24,25]. In turn, sleep disorders can worsen existing comorbidities [2]. Further longitudinal data are needed to assess the prevalence and variation of sleep disorders across the lifespan in people with DS.

On the other hand, there are differences in sleep disturbance reports among subjective measures such as sleep questionnaires and sleep diaries, and objective sleep measures such as actigraphy or polysomnography. The rates of BSD in adults with DS estimated in family reports obtained from clinical interviews and online community-based surveys ranged from 22.75% to 48% [2,5], whereas BSD rates of up to 75% were reported based on objective sleep measures and well-defined sleep criteria [14,24]. These data illustrate that sleep problems are underestimated by both parents and caregivers in DS populations [14].

#### 3.1.1. Diagnosis

The lack of validated questionnaires to screen for sleep disturbances in adults with DS might, in part, contribute to these discrepancies. Some pediatric sleep questionnaires developed for typically developing (TD) children have been used for children with DS, and the Children’s Sleep Habits Questionnaire is the one with the strongest psychometric properties [7]. However, for children with DS, questionnaires can fail to detect 66% of sleep problems [1]. In adults with DS, the only published study that compared sleep quality between subjective and objective measures lacked the sensitivity of subjective sleep measures (the Pittsburgh Sleep Quality Index) to detect sleep disorders found in 74% of polysomnography tests [1].

The objective measures to quantify sleep disruption include polysomnography (the gold standard) and actigraphy. Polysomnography studies of people with DS consistently show a high prevalence of sleep fragmentation, with longer waking after sleep onset and lower sleep efficiency (<85%) than in the TD population [14,17,26]. A characteristic DS sleep phenotype has been suggested, with decreased rapid-eye movement (REM) sleep and increased slow-wave sleep (SWS) [14,26]. Normative data on sleep electroencephalography (EEG) patterns and their evolution across the lifespan are still lacking.

An actigraph is a small watch-like device that monitors sleep and wake patterns based on motor activity 24 h/day in the subject’s habitual home environment for a period of 1–2 weeks [6,13,14]. In children with DS, actigraphy measures show good convergent validity with polysomnographic measures [27]. The validity of actigraphy has not been determined for adults with DS. Of note, as in the general population, actigraphy measures underestimate waking time during the night, resulting in higher sleep efficiency according to actigraphy compared with polysomnography [14,27]. In our recent study [14], we showed actigraphy-recorded sleep time during the day that was not captured in the sleep diaries reported by the caregiver.

#### 3.1.2. Consequences

Behavioral sleep disturbances result in short, poor-quality sleep [28]. Sleep deprivation has a negative impact on general health, daytime behavior, and cognitive function [10].

In children with DS, parental reports of sleep disturbances have been associated with more behavioral problems in school, deficits in accomplishing daily activities [29], attention deficit hyperactivity, and less effort in controlling temperament [30]. Indeed, restless sleep behavior was predictive of executive dysfunction, with poorer inhibitory control, shifting, and working memory based on reports by parents and teachers [7].

**Figure 2:**
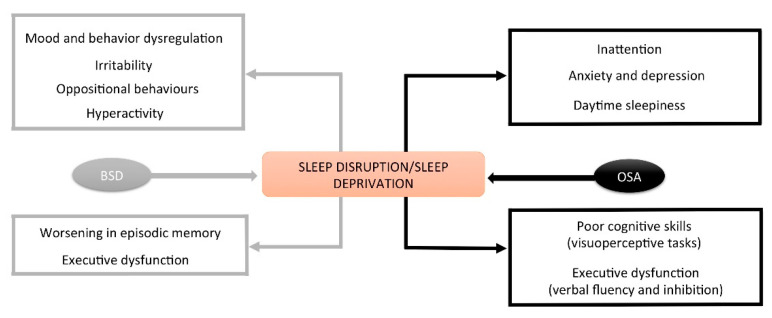
Impact of sleep behavioral disturbances and OSA on cognitive and behavioral functions.

Similar results have been reported in adults with DS [2,5] (Figure 2).

Moreover, higher levels of sleep disruption, as measured by actigraphy, have been associated with worse episodic memory, executive functioning, motor planning, and coordination [24]. Behavioral sleep disturbances have also been associated with more frequent hospital admissions and emergency department visits and vascular and mental health conditions [2,7]. Older adults with behavioral sleep problems may also be at higher risk for dementia, anxiety, or depression [2,5]. Of note, fragmented sleep in people with DS also impacts the quality of sleep of their family caregivers [31]. In a study on adults with DS, 58% of the surveyed caregivers felt that their sleep was affected, which can affect family bonds [2].

#### 3.1.3. Treatment

**Figure 3:**
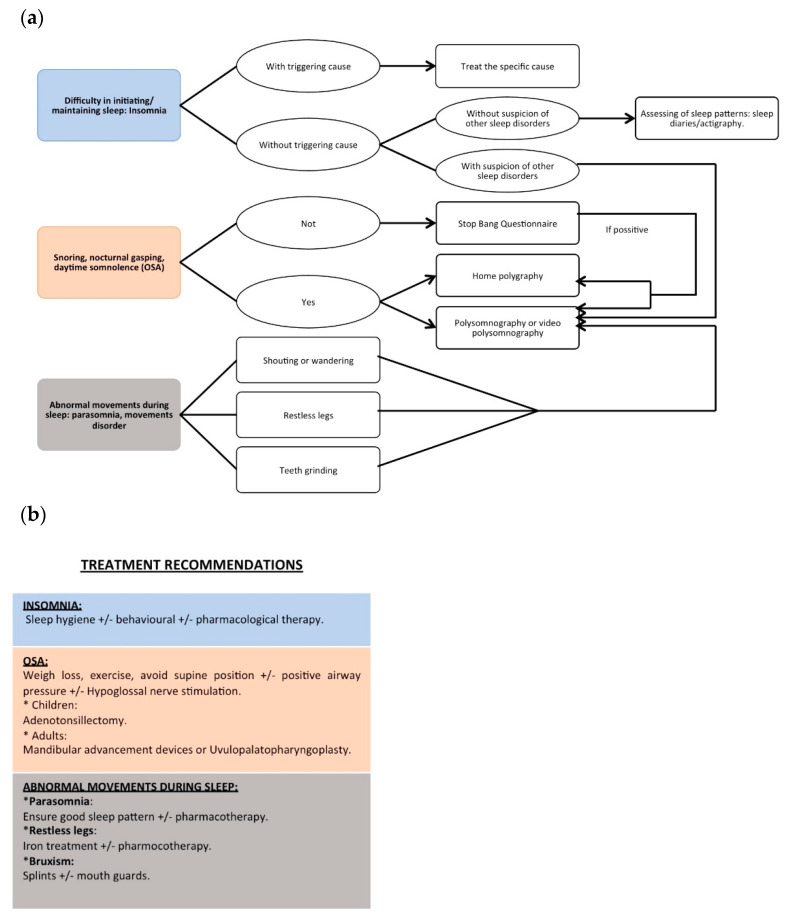
Recommendations for (**a**) diagnosis and (**b**) treatment of most frequent sleep disorders in Down syndrome.

Management of insomnia requires a gradual strategy (Figure 3). It should begin with attempts to eliminate or minimize any behavioral, psychological, environmental, and medical comorbidity that disrupt sleep. Specifically, the treatment of behavioral sleep problems is based mainly on behavior modification techniques that eliminate inappropriate behaviors before and during sleep [12]. Sleep hygiene, stimulus control techniques, and the promotion of routines that facilitate effective sleep are recommended [28]. The involvement of caregivers, especially for institutionalized subjects, is the key to success [32]. Nonetheless, there is a lack of randomized behavioral therapy trials in the DS population. There are only some case-series studies that report improvements in sleep efficiency [32,33].

Evidence of a pharmacological approach to insomnia in adults with DS is also lacking. The only medication that has been evaluated is melatonin, in a small randomized controlled trial of adults with DS [33]. At doses ranging from 2.5 to 10 mg with a delayed-release formulation, it reduced sleep latency and increased sleep efficiency [33,34].

### 3.2. Sleep Breathing Disorders

**Figure 4:**
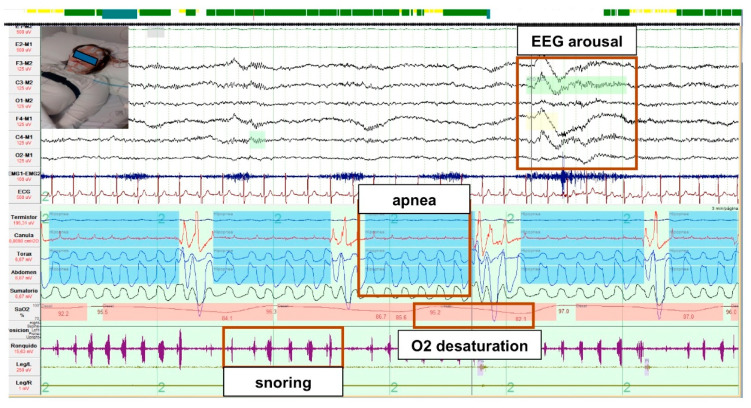
Polysomnographic recording from a patient with DS and OSA. Polysomnographic recording with five apneic events during sleep, shown by reduced airflow with preserved respiratory effort, associated with severe desaturations after airway collapse, with cortical arousals with airway reopening.

Sleep breathing disorders (SBD) include medical conditions that lead to abnormal respiration during sleep. Obstructive sleep apnea (OSA) is the most frequent sleep breathing disorder, characterized by recurrent events of partial (hypopnea) or complete (apnea) upper airway obstruction with disruption of normal oxygenation, ventilation, and sleep patterns (Figure 4) [35].

OSA is extremely common in the DS population [3,36]. The prevalence of OSA in children with DS ranges from 30 to 76% compared to 2% in TD children [37,38]. In adults with DS, it is even more frequent, with 65–100% having OSA, compared to 5–7% in TD adults [2,5,14,18,24,38].

Most sleep studies were conducted in hospital-based settings, based on referrals from DS clinics or clinical suspicion of OSA, suggesting a possible bias toward higher prevalence estimates (Table 1). Two population-based studies confirmed the high prevalence. Hill et al. [19], in a population-based survey of over 1100 adults with DS in the UK, estimated an OSA prevalence of 35% based on self-reported symptoms. Giménez et al. [14], in a community-based polysomnographic sleep study with a large cohort of adults with DS, found an OSA prevalence of 78%, which was five times higher than in the control group. Importantly, OSA was undetected by caregivers or in self-reports of sleep quality. A poor correlation between parental OSA reports and polysomnography data has also been found for children with DS [37,39].

There are multiple phenotypic and physiological factors that predispose individuals with DS to OSA [38,40], including anatomical factors such as small oropharynx with mandibular and mid-facial hypoplasia (which result in relative macroglossia and glossoptosis) [41]. Adenotonsillar hyperplasia, which narrows the upper airway structure, is also frequently found [42]. People with DS have general hypotonia and increased mucosal secretions and a higher incidence of upper respiratory tract infections and gastroesophageal reflux, all of which can impact airway flow [43]. Finally, with aging, hypothyroidism and obesity are also frequent, which additionally increase the risk for OSA [14,44].

Older age is associated with higher OSA prevalence and severity in some studies [14,21] but not in others [2]. Gender does not seem to influence OSA prevalence in adults with DS [2,14,19,21], whereas in the general population, there is a higher prevalence among males [35]. There is conflicting evidence on the impact of obesity on OSA prevalence in the DS population, with some studies showing a positive correlation between body mass index and OSA in children [45] and adults [2,17,20,21,22] but not others [14,16,19,37]. In the general population, obesity predisposes people to OSA due to excessive fat deposition in the neck and waist, but this relationship is less clear in children and adults with DS [21]. Hypothyroidism has not been systematically studied in DS [14,17].

OSA in the DS population is also more severe than in the general population, with worse apnea hypopnea index values and more hypoxemia. More than half of children [37,38] and adults with DS have severe OSA [14,20,21,22].

#### 3.2.1. Diagnosis

The American Academy of Pediatrics (AAP) recommends a baseline polysomnogram of all children with DS by age 4 years, or earlier if OSA symptoms are present [46,47]. In adults, the recommendation is to perform OSA screening, but there are no current guidelines defining how or when it should be performed [9].

Symptoms such as snoring, pauses in breathing, and restless sleep with daytime hyperactivity are suggestive of pediatric sleep apnea [37]. Complaints about bad sleep quality and nocturnal signs of OSA are in contrast rare in adults with DS [37]. Excessive napping, morning fatigue, humor or behavioral changes, and poor ability to concentrate are the predominant daytime OSA signs [2,19]. Questionnaires to assess OSA have not been validated for adults with DS [40]. The STOP-BANG score has been recommended to detect moderate to severe OSA [22]. The Epworth Sleepiness Scale (ESS) and its pictorial form (pESS) [48] have low sensitivity but are the most widely used tests to assess sleepiness [2,14,17,19,22]. It remains to be proven whether adjusting questions on the ESS to the particular abilities and lifestyles of individuals with DS could improve OSA detection [48]. In this sense, Skotko et al. [45] proposed an algorithm model that could predict which patients with DS were unlikely to have severe OSA, in order to avoid unnecessary sleep studies. However, nowadays, due to the unreliability of parental reports and questionnaires, diagnostic OSA confirmation requires overnight testing (Figure 3).

Polysomnography (PSG) is the gold standard to diagnose sleep disorders and OSA, as it monitors sleep and respiratory parameters overnight (Figure 4). It requires the placement of several leads, which can make the test difficult to conduct [35]. This has raised questions about the feasibility of conducting successful polysomnography in the DS population [2,19,21]. However, Hurvitz et al. [49], in a recent large study with 248 children with DS, showed that 96% were able to tolerate and complete a valid polysomnography test. PSG seems to be equally well tolerated by adults with DS, [14,20,24]. Providing an additional night for adaptation improves tolerance to polysomnography, but does not increase sleep efficiency [14].

There are alternative diagnostic methods to hospital polysomnography studies. Home sleep apnea testing with polygraphy or oximetry uses devices that monitor respiratory parameters but cannot obtain sleep parameters [35]. The sensors are self-applied by the patient at home. These ambulatory devices have been successfully used in DS [15,19,21]. Polygraphy for children with DS shows a slightly lower OSA prevalence than polysomnography [38]. Sensitivity and specificity of polygraphy were higher when diagnosing moderate to severe OSA [50]. Validation of this test in adults with DS has not yet been performed. Oximetry seems to have poor sensitivity for OSA screening in the DS population [40].

#### 3.2.2. Treatment

Effective treatments for OSA include behavioral measures, surgery, and medical devices [51] (Figure 3). Deciding on the most effective treatment will depend (as in the general population) on disease severity, presented symptoms, and contributing causes, as well as, importantly in the case of the DS population, on caregiver support.

Behavioral measures and lifestyle modifications include abstaining from alcohol, avoiding the supine sleep position, engaging in regular aerobic exercise, and losing weight [35]. In the general population, any weight loss intervention (lifestyle, bariatric surgery, or medication) improves OSA severity in a dose-dependent manner [52]. Exercise may improve OSA independently of weight loss [53]. In DS, exercise improves sleep duration and sleep quality in obese subjects [43,53], which improves motor dysfunction and exercise tolerance [43]. Multidisciplinary weight loss interventions are also recommended for individuals with DS [54], but there are no sleep studies available that objectively assess the effect of weight loss on sleep apnea severity.

The most common surgical procedure for managing OSA is modification of the upper airway soft tissue. Adenotonsillectomy is the first line treatment for pediatric OSA [47], with 80% OSA resolution in TD children [55]. In children with DS, adenotonsillectomy is effective in reducing OSA severity [49,55] but with higher OSA persistence (ranging from 50 to 75% after surgery) [49,56,57]. Obesity is the major risk factor associated with persistent OSA in TD children [38], but it might not be as important in children with DS [37,49,55], probably due to the multifactorial nature of the upper airway obstruction in this population [42].

Further surgical options currently include tongue base surgery, uvulopalatopharyngoplasty, oromaxillary surgery, and tracheostomy in severe cases [51]. These options are less used because they pose varying degrees of risk and are associated with more complications in the DS population due to underlying comorbidities [43,55].

Hypoglossal nerve stimulation (HNS) is a novel OSA surgical procedure in which an electrode is implanted that stimulates the hypoglossal nerve to enhance tongue protrusion and stabilize the upper airway during inspiration [58]. Recent small studies suggest that hypoglossal stimulation could also be effective and safe in the DS population. In a study of 20 adolescents with DS and residual severe OSA, hypoglossal stimulation reduced the apnea hypopnea index in 85% without long-term complications and median nightly usage of 9.21 h after 12 months of follow-up [59]. Similar results were reported for adults with DS, with good rates of HNS efficacy, good device adherence, low surgical complication rates, and reported subjective improvements in sleep quality [60].

Continuous positive airway pressure (CPAP) is the medical technique used in routine clinical practice for persistent OSA in children [55,61] and the primary therapy for adults with moderate to severe or symptomatic OSA [35]. A CPAP device delivers pressure to the airway through a mask worn over the nose or the nose and mouth and prevents airway collapse during inspiration. The effectiveness of CPAP treatment, however, is contingent on adherence to the therapy [62]. Poor tolerance with insufficient use is reported for almost one-third of TD OSA patients [63]. Acceptable rates of good acceptance and efficacy of the treatment have been reported for children with DS, especially older children [61].

In adults with DS, despite the high prevalence of severe OSA, CPAP treatment is not usually proposed. It is often presumed that they will not tolerate the treatment [14,23,64]. Data on CPAP use by adults with DS are therefore very limited (Table 1). Three of the four published studies on objective CPAP use reported good compliance (>4 h/night). Prospective studies show good compliance in 60–80% of subjects after 6 months and 1 year of treatment [17,65,66]. We recently reported good compliance by 81% of OSA patients with DS after 3 years of follow-up [66]. Retrospective data have also shown good CPAP compliance by 79% of subjects [21], with clinical subjective improvement in daytime function and alertness [17,21]. On the contrary, insufficient CPAP compliance (<4 h/night) was found in one prospective study after 1 year of follow-up [23]. This study had a two phase-design, a 1 month randomized controlled pilot trial with a posterior 12-month cohort follow-up study, incorporating repeated measures of sleepiness, behavior, and general health. In the initial CPAP trial at 1 month, changes between groups were not observed. In the posterior evaluation after 12 months of treatment, despite the low CPAP use, with a mean of 1.5 h/night, significant decreases were noted in somnolence (pESS), anxiety, and depressive behavior scores, with improvement in intelligence and general health scores compared to baseline scores before CPAP treatment [23]. Disease factors such as baseline OSA severity and excessive daytime somnolence have not been associated with better long-term CPAP compliance by adults with OSA and DS [14,21].

These studies globally show the feasibility of CPAP treatment and generally good compliance by adults with DS and OSA. Most of these studies used an intensive CPAP training protocol to increase compliance [17,21,65], but we reported a good long-term compliance using the same clinical protocol routinely used in the general population [66]. Despite the good adherence, there are no standardized criteria or evidence-based guidelines regarding CPAP titration for children or adults with DS. The effects of CPAP treatment on comorbidities have not been explored. Randomized controlled trials are warranted to determine the efficacy of CPAP treatment on reducing adverse OSA consequences in this population.

Oral appliances are devices that protrude and advance the mandible relative to the maxilla, resulting in enhanced volume and consequently reducing upper airway collapsibility during sleep. They are increasingly used to treat mild to moderate OSA in the general population, especially for people who do not tolerate CPAP treatment [67]. There are only some anecdotal clinical cases published on adults with DS. These cases show good efficacy in decreasing respiratory events, although they are based on caregiver observations [68]. Characteristic anatomical features of the DS phenotype, such as (relative) macroglossia or micrognathia, may be obstacles to tolerating such appliances. Fortunately, clinical trials to demonstrate the feasibility and efficacy of these devices in the DS population are being investigated [69].

#### 3.2.3. Consequences of OSA

OSA is a systemic disorder that affects cardiovascular and metabolic systems, as well as cognitive functioning [35]. Due to the cognitive and medical comorbidities associated with DS, the consequences of OSA may have a greater impact in this population.

##### Cognitive

OSA in the general population results in cognitive deficits, particularly in the domains of attention, vigilance, psychomotor function, language–memory, and executive functioning [70]. Similarly, it can be presumed that the consequences of OSA affect the cognitive performance of children with DS [71]. Some studies of children with DS and OSA have shown lower verbal IQ scores, worse performance on executive tasks [72] and communications skills, and greater daytime somnolence [29] compared to children without OSA. Other studies, however, have not found such differences [73,74].

In adults with DS, the effects of OSA on cognition have been less studied, but also suggest an impact on cognitive skills and executive functioning. In a study of 29 young adults, parental ratings of OSA severity were associated with poorer verbal fluency and inhibition and daytime sleepiness with poor inhibitory control [18]. In an objective sleep study with home polygraphy, more apneas per hour was associated with worse visuoperceptual skills [18].

Cognitive deficits associated with OSA may arise from direct neural damage to frontal and hippocampal regions as a result of intermittent hypoxia and changes in cerebral blood flow, or as a consequence of OSA-induced sleepiness or reduced psychomotor speed [13]. These cognitive disturbances are associated with neuroanatomical changes in frontal, parietal, temporal, and hippocampal regions [75]. The neurocognitive profile of people with DS is also characterized by prefrontal cortex and hippocampus dysfunction [76]. These data suggest that there is an overlap of neurocognitive dysfunctions between DS and OSA, especially in executive functions [46].

Growing evidence suggests that there is a link between sleep disturbances, cognitive impairment, and Alzheimer’s disease [13,77,78]. Sleep disruption increases amyloid accumulation due to increased production during wakefulness and decreased clearance in sleep, as measured by CSF, blood, and PET biomarkers [77,78]. DS is a genetic form of Alzheimer’s disease with a long and predictable preclinical phase similar to that in the sporadic and autosomal dominant form [11]. The triplication of the amyloid precursor protein (APP) located in chromosome 21 increases the production of amyloid beta (Aβ). The lifetime risk for early-onset Alzheimer’s disease is over 90% [11,79]. OSA might be a modifiable risk factor for cognitive decline and AD dementia. Only one study has examined the relationship between sleep and AD biomarkers in DS. Cody et al. [24] studied cognitive functioning in 47 adults with DS and measured sleep with actigraphy and beta amyloid with Pittsburgh Compound B positron emission tomography imaging. The length of nighttime awakenings was significantly associated with a higher striatal beta amyloid burden and worse performance in executive functioning, motor planning, and coordination measures. A limitation of that study was the absence of objective sleep measures to screen for OSA and sleep stages. Further PSG, multimodal, and longitudinal studies are needed to evaluate the relationship between OSA, cognitive performance, and AD in DS.

##### Cardiovascular

In the TD population, OSA increases the risk of developing comorbid cardiovascular disease and leads to worse outcomes [35]. In contrast, the cardiovascular risk profile of people with DS is different than that of the general population, with less hypertension, and with anecdotal evidence of atherosclerosis [80]. There are no studies assessing cardiovascular outcomes in adults with DS. Very few studies have assessed the cardiovascular effects of OSA in children with DS, but showed a dampened sympathetic response to OSA [81]. An improvement in left ventricular diastolic function after CPAP use was suggested [82].

Besides cognitive and vascular consequences, other health problems are associated with OSA but have not yet been investigated in the DS population [83]. There is a relationship between sleep disorders and mood problems in adults with DS that can manifest as new onset mood disorders or declining adaptive skills, increasing the frequency of OSA diagnosis [9]. The relationship between epilepsy and OSA, especially in adulthood, warrants investigation [84].

### 3.3. Sleep Movement Disorders

Children with Down syndrome present abnormal movements during sleep that include sleep talking (19%), bruxism (17%), head banging (7%), and sleepwalking (3%) [1,6]. In turn, the concomitant high prevalence of OSA in these children can trigger arousal disorders such as sleepwalking, sleep terrors, and confusional arousals. However, all of these data were inferred from parent reports, which probably underestimate prevalence, as in other sleep disorders. In a recent objective study performed with young adults with DS, the prevalence of bruxism was 13.1% according to parent reports, in contrast to the 91.3% prevalence measured by PSG [85]. On the other hand, in a recent retrospective study of 418 children with DS who underwent polysomnography in a sleep center, only 13 subjects (3%) were referred due to concerns about restless leg syndrome (RLS) or periodic limb movement disorder (PLMS); 139 of the children (33.3%) presented PLMS, and 36 (55.4%) of them had ferritin levels under normal values (<50 ng/mL). PLMS causes sleep disturbances and is therefore an unnoticed factor with detrimental effects on daytime function, behavior, and cognition [86]. As in TD children, PLMS treatment with supplemental iron in children with DS and low serum ferritin levels could improve sleep quality and cognition [87].

In adults with DS, only one study has evaluated this problem by full videopolysomnography, a technique to score movement disorders during sleep [14]. In our recent study [14], we observed sleep talking and repetitive swallowing and chewing movements, but without bruxism features, and a large amount of leg movements (mostly accommodation movements, without meeting the criteria of PLMS). Despite the relative increase in slow wave sleep, we found no episodes of parasomnia or REM sleep behavior disorder related to non-rapid eye movement in the short periods of REM sleep recorded.

## 4. Future Directions

There are several important clinical and research gaps in knowledge of sleep disorders in adults with DS [83]. (Table 2 and Table 3).

Clinically, efforts to increase education regarding the importance of sleep problems screening in adults with DS and to validate diagnostic tools are needed. Sleep guidelines to schedule routine polysomnography screening for OSA, its treatment and ongoing surveillance in adults with DS need further development. Moreover, longitudinal data will warrant the assessment of the prevalence and variation of sleep disorders across the lifespan of people with DS. From a research perspective, additional longitudinal, multimodal and population-based studies will help to identify risk factors and assess consequences of sleep disorders. Clinical randomized and control trials will be of added value to determine the efficacy of the different treatment approaches in this population.

## 5. Conclusions

Sleep disorders are very frequent in the DS population, with a higher prevalence of obstructive sleep apnea and insomnia than in the general population. However, especially in adulthood, they are underrecognized and attributed to the aging process in DS. Untreated sleep disruption impairs behavior and cognitive function and probably accelerates progression to AD dementia. There are important educational, clinical, and research gaps that should be addressed in order to reach an earlier diagnosis. The effective treatment of sleep disorders in adults with DS is a very important goal to preserve their full potential in terms of mental and physical health and quality of life as they age.

## Figures and Tables

**Table 1:** Salient sleep studies of adults with DS.

Citation	Design	Population (Women %)	Age	Sleep Study	Outcomes
Andreu et al. (2002) [15]	Prospective DS clinic	DS: 12 (50%)	21.6 (17–31) y	Polygraphy	OSA associated with worse visuoperceptual skills
Resta et al. (2003) [16]	Prospective DS clinic	DS: 6 (50%)	38.6 (28–53) y	PSG	83% OSA No relation with age neither BMI
Trois et al. (2009) [17]	Prospective DS clinic	DS: 16 (50%) TD: 48	33 (19–56) y	PSG	94% OSA 69% severe OSA AHI-BMI correlation 62% CPAP (7 h/night)
Chen et al. (2013) [18]	Prospective DS clinic	DS: 29 (27.6%)	20.26 (14–31)y	Sleep reports	OSA severity related to executive dysfunction
Hill (2016) [19]	Prospective (a) Population-based survey	DS (a) 1100	>16 y	(a) pEES	(a) 35% OSA self reporting symptoms
	(b) Sleep center OSAsuspected	(b) 134	>16 y	(b) home Poligraphy	(b) 42% OSA (AHI > 10)
Esbensen (2016) [5]	Prospective DS clinic	DS: 75 (34.7%)	51.1 (37–65) y	Clinical interview	22.4% BSD, poorer health 13% OSA, more visits to physicians
Giménez et al. (2018) [6]	Prospective Community sample	DS: 54 (38.3%) TD: 35	39.6 (20–62) y	PSG, actigraphy, ESS, PSQI, BQ	74% Sleep disruption 78% OSA (44.1% severe), both undetected by self-reported measures
Cornacchia et al. (2019) [20]	Retrospective DS clinic	DS: 125 (39.9%)	28.8 (18–62) y	PSG	82.1% OSA 39.1% severe OSA
Stores (2019) [2]	Prospective DS clinic	DS: 100 (45%)	28 y (16–61) y	Online survey	25% OSA, 48% BSD ESS:8.5 Yes: OSA-BMI, osa-ESS58% Caregiver’s sleep affected
Landete et al. (2020) [21]	Retrospective Sleep center OSAsuspected	DS: 114 (40.7%)	36 y	91% poligraphy 9% PSG	72.6% OSA (53.5% severe) CPAP use 65% (7 h/day)
Carvalho et al. (2020) [22]	Prospective DS clinic	DS: 60 (45%)	27 y	Home polygraphy ESS, PSQI, BQ, SBQ	100% OSA (38.3% severe) SBQ good sensitivity and OSA specificity
Hill et al. (2020) [23]	1 month prospective CPAP Trial Follow up 1y	DS: 29 (32%)	28 y	Home PSG pESS; Cognitive measures	68% use CPAP (2.8 h/night) after 12 months follow up Any differences after 1 m, but after a 12 m
Cody et al. (2020) [24]	Prospective DS clinic	DS: 47 (51.1%)	38.4 (26–56) y	Actigraphy, PIB, PET, Cognitionassessment	Longer awakenings associated with an increase striatal amyloid burden and decreased cognition

DS, Down syndrome; TD, typically developing control; ESS, Epworth Sleepiness Scale; pESS, pictorical Epworth Sleepiness Scale; PSQ, Pittsburgh Sleep Quality Index; BQ, Berlin; SBQ, STOP-BANG questionnaires; PSG, polysomnography; CPAP, continuous positive airway pressure; OSA, obstructive sleep apnea; BMI, body mass index.

**Table 2:** Gaps and recommendations for sleep clinical assessment.

Clinical Gaps	Practical Recommendations
Sleep problems are underreported	Enhance sleep education for caregivers to Increase awareness of the importance of maintaining good sleep as an essential factor of healthy aging - Encourage seeking medical help after any sleep changes - Realize that sleep problems may be a red flag indicating other underlying health problems
Sleep disorders are overlooked and underdiagnosed by health care professionals	Perform structured anamnesis to screen for sleep problems at every routine medical visit
There is a high prevalence of asymptomatic OSA in people with DS of all ages	Schedule polygraphy or polysomnography and surveillance across lifespan
Referral for polysomnography is poor due to fear or presuming the subject would not tolerate the test	Provide detailed explanations of polysomnography procedures and reassurance based on objective feasibility data
There is skepticism about the feasibilities and benefits of sleep theraphies, as CPAP treatment, for adults with DS	Promote earlier treatment of sleep disorders to prevent health and cognitive consequences. Sleep disorders can worsen other comorbidities.

**Table 3:** Research gaps and methodological research recommendations.

Research Gaps	Methodological Research Recommendations
There is a lack of validated sleep questionnaires Validation of portable sleep screening devices is needed Recommendations for screening, treatment, and following up sleep disorders are needed	Design sleep scales appropriate for adults with DS Measure objective accuracy by comparing with polisomnography data Develop mandatory sleep guidelines for adults with DS
There is a lack of normative data on sleep EEG and sleep wake patterns Data of sleep problems over time in different age groups should be correlated Comorbidities and risk factors for sleep disorders and predictive factors of treatment adherence should be identified	Perform longitudinal polisomnography and actigraphy studies across childhood, adulthood, and old age
Most available sleep studies focused on OSA	Conduct further research on other sleep disorders such as circadian rhythm disorders
There is a lack of objective data on the efficacy of sleep disorder treatment procedures	Conduct randomized control clinical trials
In a majority of studies subjects were referred due to sleep problems	Conduct population-based studies
Few cognitive studies have objective sleep data and assess relationships with other objective neurological markers	Design multimodal studies with objective sleep evaluation, DS-specific cognitive battery, imaging, and AD biomarkers

## Data Availability

Data are available on reasonable request. The data generated and analysed during the present study are under the domain of the corresponding author and will be made available on request and evaluation.

## References

[1] Hoffmire C.A., Magyar C.I., Connolly H.V., Fernandez I.D., Van Wijngaarden E. (2014). High Prevalence of Sleep Disorders and Associated Comorbidities in a Community Sample of Children with Down Syndrome. J. Clin. Sleep Med..

[2] Stores R.J. (2019). Sleep Problems in Adults with Down Syndrome and Their Family Carers. J. Appl. Res. Intellect. Disabil..

[3] Smith D.S. (2001). Health care management of adults with Down syndrome. Am. Fam. Physician.

[4] Carter M., McCaughey E., Annaz D., Hill C.M. (2008). Sleep Problems in a Down Syndrome Population. Arch. Dis. Child..

[5] Esbensen A.J. (2016). Sleep Problems and Associated Comorbidities among Adults with Down Syndrome. J. Intellect. Disabil. Res..

[6] Stores G., Stores R. (2012). Sleep Disorders and Their Clinical Significance in Children with Down Syndrome. Dev. Med. Child Neurol..

[7] Esbensen A.J., Hoffman E.K. (2016). Reliability of Parent Report Measures of Sleep in Children with Down Syndrome. J. Intellect. Disabil. Res..

[8] Bittles A.H., Glasson E.J. (2007). Clinical, Social, and Ethical Implications of Changing Life Expectancy in Down Syndrome. Dev. Med. Child Neurol..

[9] Capone G.T., Chicoine B., Bulova P., Stephens M., Hart S., Crissman B., Videlefsky A., Myers K., Roizen N., Esbensen A. (2017). Co-Occurring Medical Conditions in Adults with Down Syndrome: A Systematic Review toward the Development of Health Care Guidelines. Am. J. Med. Genet..

[10] Medic G., Wille M., Hemels M.E. (2017). Short- and Long-Term Health Consequences of Sleep Disruption. Nat. Sci. Sleep.

[11] Fortea J., Vilaplana E., Carmona-Iragui M., Benejam B., Videla L., Barroeta I., Fernández S., Altuna-Azkargorta M., Pegueroles J., Montal V. (2020). Clinical and Biomarker Changes of Alzheimer’s Disease in Adults with Down Syndrome: A Cross-Sectional Study. Lancet.

[12] Riemann D., Baglioni C., Bassetti C., Bjorvatn B., Groselj L.D., Ellis J.G., Espie C.A., Garcia-Borreguero D., Gjerstad M., Gonçalves M. (2017). European Guideline for the Diagnosis and Treatment of Insomnia. J. Sleep Res..

[13] Cedernaes J., Osorio R.S., Varga A.W., Kam K., Schiöth H.B., Benedict C. (2017). Candidate Mechanisms Underlying the Association between Sleep-Wake Disruptions and Alzheimer’s Disease. Sleep Med. Rev..

[14] Giménez S., Videla L., Romero S., Benejam B., Clos S., Fernández S., Martínez M., Carmona-Iragui M., Antonijoan R.M., Mayos M. (2018). Prevalence of Sleep Disorders in Adults with Down Syndrome: A Comparative Study of Self-Reported, Actigraphic, and Polysomnographic Findings. J. Clin. Sleep Med..

[15] Andreou G., Galanopoulou C., Gourgoulianis K., Karapetsas A., Molyvdas P. (2002). Cognitive Status in Down Syndrome Individuals with Sleep Disordered Breathing Deficits (SDB). Brain Cogn..

[16] Resta O., Barbaro M.P.F., Giliberti T., Caratozzolo G., Cagnazzo M., Scarpelli F., Nocerino M. (2003). Sleep Related Breathing Disorders in Adults with Down Syndrome. Down Syndr. Res. Pr..

[17] Trois M.S., Capone G.T., Lutz J.A., Melendres M.C., Schwartz A.R., Collop N.A., Marcus C.L. (2009). Obstructive Sleep Apnea in Adults with Down Syndrome. J. Clin. Sleep. Med..

[18] Chen C.-C., Spanò G., Edgin J. (2013). The Impact of Sleep Disruption on Executive Function in Down Syndrome. Res. Dev. Disabil..

[19] Hill E.A. (2016). Obstructivesleep Apnoea/Hypopnea Syndrome in Adults with Down Syndrome. Breathe.

[20] Cornacchia M., Sethness J., Alapat P., Lin Y.-H., Peacock C. (2019). The Prevalence of OSA Among an Adult Population with Down Syndrome Referred to a Medical Clinic. Am. J. Intellect. Dev. Disabil..

[21] Landete P., Soriano J.B., Aldave B., Zamora E., Acosta C., Erro M., Riolobos C.L., Ramos M.I., Moldenhauer F., Ancochea J. (2020). Obstructive Sleep Apnea in Adults with Down Syndrome. Am. J. Med Genet. Part A.

[22] Carvalho A.A., Amorim F.F., Santana L.A., Almeida K.J.Q., Santana A.N.C., Neves F.A.R. (2020). STOP-Bang Question-Naire Should be Used in All Adults with Down Syndrome to Screen Formoderate to Severe Obstructive Sleep Apnea. PLoS ONE.

[23] Hill E.A., Fairley D.M., Williams L.J., Spanò G., Cooper S.-A., Riha R.L. (2020). Prospective Trial of CPAP in Community-Dwelling Adults with Down Syndrome and Obstructive Sleep Apnea Syndrome. Brain Sci..

[24] Cody K.A., Piro-Gambetti B., Zammit M.D., Christian B.T., Handen B.L., Klunk W.E., Zaman S., Johnson S.C., Plante D.T., Hartley S.L. (2020). Association of Sleep with Cognition and Beta Amyloid Accumulation in Adults with Down Syndrome. Neurobiol. Aging.

[25] Startin C.M., D’Souza H., Ball G., Hamburg S., Hithersay R., Hughes K.M.O., Massand E., Karmiloff-Smith A., Thomas M.S.C., Consortium L. (2020). Health Comorbidities and Cognitive Abilities Across the Lifespan in Down Syndrome. J. Neurodev. Disord..

[26] Nisbet L.C., Phillips N.N., Hoban T.F., O’Brien L.M. (2014). Characterization of a Sleep Architectural Phenotype in Children with Down Syndrome. Sleep Breath.

[27] Esbensen A.J., Hoffman E.K., Stansberry E., Shaffer R. (2018). Convergent Validity of Actigraphy with Polysomnography and Parent Reports when Measuring Sleep in Children with Down Syndrome. J. Intellect. Disabil. Res..

[28] Turnbull K., Reid G.J., Morton J.B. (2013). Behavioral Sleep Problems and their Potential Impact on Developing Executive Function in Children. Sleep.

[29] Churchill S.S., Kieckhefer G.M., Bjornson K.F., Herting J.R. (2015). Relationship between Sleep Disturbance and Functional Outcomes in Daily Life Habits of Children with Down Syndrome. Sleep.

[30] Lukowski A.F., Milojevich H.M. (2016). Sleep Problems and Temperament in Young Children with Down Syndrome and Typically Developing Controls. J. Intellect. Disabil. Res..

[31] Gallagher S., Whittaker A., Carroll D. (2009). Parental Stress is Associated with Poor Sleep Quality in Parents Caring for Children with Developmental Disabilities. J. Pediatr. Psychol..

[32] Van De Wouw E., Evenhuis H., Echteld M. (2012). Prevalence, Associated Factors and Treatment of Sleep Problems in Adults with Intellectual Disability: A Systematic Review. Res. Dev. Disabil..

[33] Shanahan P.J., Palod S., Smith K.J., Fife-Schaw C., Mirza N. (2019). Interventions for Sleep Difficulties in Adults with an Intellectual Disability: A Systematic Review. J. Intellect. Disabil. Res..

[34] Ward F., Nanjappa M., Hinder S.A.J., Roy M. (2015). Use of Melatonin for Sleep Disturbance in a Large Intellectual Disability Psychiatry Service. Int. J. Dev. Disabil..

[35] Gottlieb D.J., Punjabi N.M. (2020). Diagnosis and Management of Obstructive Sleep Apnea: A Review. JAMA.

[36] Simson R., Oyekan A.A., Ehsan Z., Ingram D.G. (2018). Obstructive Sleep Apnea in Patients with Down Syndrome: Current Perspec-tives. Nat. Sci. Sleep.

[37] Maris M., Verhulst S., Wojciechowski M., Van de Heyning P., Boudewyns A. (2016). Sleep Problems and Obstructive Sleep Apnea in Children with Down Syndrome, an Overview. Int. J. Pediatr. Otorhinolaryngol..

[38] Lee C.-H., Hsueh W.-Y., Lin M.-T., Kang K.-T. (2018). Prevalence of Obstructive Sleep Apnea in Children with Down Syndrome: A Meta-Analysis. J. Clin. Sleep Med..

[39] Rosen D., Lombardo A., Skotko B., Davidson E.J. (2010). Parental Perceptions of Sleep Disturbances and Sleep-Disordered Breathing in Children with Down Syndrome. Clin. Pediatr..

[40] Lal C., White D.R., Joseph J.E., van Bakergem K., LaRosa A. (2015). Sleep-Disordered Breathing in Down Syndrome. Chest.

[41] Ferrario V.F., Dellavia C., Zanotti G., Sforza C. (2004). Soft Tissue Facial Anthropometry in Down Syndrome Subjects. J. Craniofacial Surg..

[42] Farhood Z., Isley J.W., Ong A.A., Nguyen S.A., Camilon T.J., LaRosa A.C., White D.R. (2017). Adenotonsillectomy Outcomes in Patients with Down Syndrome and Obstructive Sleep Apnea. Laryngoscope.

[43] Dumortier L., Bricout V.-A. (2020). Obstructive Sleep Apnea Syndrome in Adults with Down Syndrome: Causes and Consequences. Is It a “Chicken and Egg” Question?. Neurosci. Biobehav. Rev..

[44] Amr N.H. (2018). Thyroid disorders in subjects with Down syndrome: An update. Acta Biomed..

[45] Skotko B.G., Macklin E.A., Muselli M., Voelz L., McDonough M.E., Davidson E., Allareddy V., Jayaratne Y., Bruun R., Ching N. (2017). A Predictive Model for Obstructive Sleep Apnea and Down Syndrome. Am. J. Med. Genet. Part A.

[46] Bull M.J. (2011). The Committee on Genetics Health Supervision for Children with Down Syndrome. Pediatrics.

[47] Marcus C.L., Brooks L.J., Draper K.A., Gozal D., Halbower A.C., Jones J., Schechter M.S., Sheldon S.H., Spruyt K., Ward S.D. (2012). Diagnosis and Management of Childhood Obstructive Sleep Apnea Syndrome. Pediatrics.

[48] Hill E., Fairley D.M., McConnell E., Morrison I., Celmina M., Kotoulas S.-C., Riha R.L. (2020). Utility of the Pictorial Epworth Sleepiness Scale in the Adult Down Syndrome Population. Sleep Med..

[49] Hurvitz M.S., Lesser D.J., Dever G., Celso J., Bhattacharjee R. (2020). Findings of Routine Nocturnal Polysomnography in Children with DOWN Syndrome: A Retrospective Cohort Study. Sleep Med..

[50] Ikizoglu N.B., Kiyan E., Polat B., Ay P., Karadag B., Ersu R. (2019). Are Home Sleep Studies Useful in Diagnosing Obstructive Sleep Apnea in Children with Down Syndrome?. Pediatr. Pulmonol..

[51] Qaseem A., Holty J.E., Owens D.K., Dallas P., Starkey M., Shekelle P. (2013). Management of obstructive sleep apnea in adults: A clinical practice guideline from the American College of Physicians. Ann. Intern. Med..

[52] Roche J., Isacco L., Masurier J., Pereira B., Mougin F., Chaput J.-P., Thivel D. (2020). Are Obstructive Sleep Apnea and Sleep Improved in Response to Multidisciplinary Weight Loss Interventions in Youth with Obesity? A Systematic Review and Meta-analysis. Int. J. Obes..

[53] Rimmer J.H., Heller T., Wang E., Valerio I. (2004). Improvements in Physical Fitness in Adults with Down Syndrome. Am. J. Ment. Retard..

[54] Martínez-Espinosa R.M., Vila M.D.M., García-Galbis M.R. (2020). Evidences from Clinical Trials in Down Syndrome: Diet, Exercise and Body Composition. Int. J. Environ. Res. Public Health.

[55] Dudoignon B., Amaddeo A., Frapin A. (2017). Obstructive Sleep Apnea in Down Syndrome: Benefits of Surgery and Noninva-Sive Respiratory Support. Am. J. Med. Genet. A.

[56] Maris M., Verhulst S., Wojciechowski M., Van de Heyning P., Boudewyns A. (2016). Outcome of Adenotonsillectomy in Children with Down Syndrome and Obstructive Sleep Apnoea. Arch. Dis. Child..

[57] Nehme J., Laberge R., Pothos M., Barrowman N., Hoey L., Kukko M., Monsour A., Katz S.L. (2019). Treatment and Persistence/Recurrence of Sleep-Disordered Breathing in Children with Down Syndrome. Pediatr. Pulmonol..

[58] Woodson B.T., Strohl K.P., Soose R.J., Gillespie M.B., Maurer J.T., De Vries N., Padhya T.A., Badr M.S., Lin H.-S., Vanderveken O.M. (2018). Upper Airway Stimulation for Obstructive Sleep Apnea: 5-Year Outcomes. Otolaryngol. Neck Surg..

[59] Caloway C.L., Diercks G.R., Keamy D., De Guzman V., Soose R., Raol N., Shott S.R., Ishman S.L., Hartnick C.J. (2020). Update on Hypoglossal Nerve Stimulation in Children with Down Syndrome and Obstructive Sleep Apnea. Laryngoscope.

[60] Li C., Boon M., Ishman S.L., Suurna M.V. (2019). Hypoglossal Nerve Stimulation in Three Adults with Down Syndrome and Severe Obstructive Sleep Apnea. Laryngoscope.

[61] Kang E.K., Xanthopoulos M.S., Kim J.Y., Arevalo C., Shults J., Beck S.E., Marcus C.L., Tapia I.E. (2019). Adherence to Positive Airway Pressure for the Treatment of Obstructive Sleep Apnea in Children with Developmental Disabilities. J. Clin. Sleep Med..

[62] Patil S.P., Ayappa I.A., Caples S.M., Kimoff R.J., Patel S., Harrod C.G. (2019). Treatment of Adult Obstructive Sleep Apnea with Positive Airway Pressure: An American Academy of Sleep Medicine Systematic Review, Meta-Analysis, and GRADE Assessment. J. Clin. Sleep Med..

[63] Cistulli P.A., Armitstead J., Pépin J.L., Woehrle H., Nunez C.M., Benjafield A., Malhotra A. (2019). Short-Term CPAP Adherence in Obstructive Sleep Apnea: A Big Data Analysis Using Real World Data. Sleep Med..

[64] Määttä T., Määttä J., Tervo-Määttä T., Taanila A., Kaski M., Iivanainen M. (2011). Healthcare and guidelines: A population-based survey of recorded medical problems and health surveillance for people with Down syndrome. J. Intellect. Dev. Disabil..

[65] Luijks K.A., Vandenbussche N.N., Pevernagie D.D., Overeem S.S., Pillen S. (2017). Adherence to Continuous Positive Airway Pressure in Adults with an Intellectual Disability. Sleep Med..

[66] Giménez S., Farré A., Morante F., Videla L., Carreras F., Benejam B., Asensio A., Carmona-Iragui M., Fortuna A., Peñacoba P. Feasibility of Continuos Airway Pressure Treatment for Sleep Apnea in Adults with Down Syndrome. Proceedings of the 3rd Internationla Conference Trisomy 21 Reserach Society.

[67] Ramar K., Dort L.C., Katz S.G., Lettieri C.J., Harrod C.G., Thomas S.M., Chervin R.D. (2015). Clinical Practice Guideline for the Treatment of Obstructive Sleep Apnea and Snoring with Oral Appliance Therapy: An Update for 2015. J. Clin. Sleep Med..

[68] Demko B.G. (2014). Oral Appliance Treatment in a Patient with Down Syndrome. J. Dent. Sleep Med..

[69] Giannasi L.C., Dutra M.T.S., Tenguan V.L.S., Mancilha G.P., Silva G.R.C., Fillietaz-Bacigalupo E., Da Silva D.B., Politti F., Nacif S.R., De Oliveira E.F. (2019). Evaluation of the Masticatory Muscle Function, Physiological Sleep Variables, and Salivary Parameters after Electromechanical Therapeutic Approaches in Adult Patients with Down Syndrome: A Randomized Controlled Clinical Trial. Trials.

[70] Leng Y., McEvoy C., Allen I.E., Yaffe K. (2017). Association of Sleep-Disordered Breathing with Cognitive Function and Risk of Cognitive Impairment. JAMA Neurol..

[71] Horne R.S., Wijayaratne P., Nixon G.M., Walter L.M. (2019). Sleep and Sleep Disordered Breathing in Children with Down Syn-drome: Effects on Behaviour, Neurocognition and the Cardiovascular System. Sleep Med. Rev..

[72] Breslin J., Spanò G., Bootzin R., Anand P., Nadel L., Edgin J. (2014). Obstructive Sleep Apnea Syndrome and Cognition in Down Syndrome. Dev. Med. Child Neurol..

[73] Brooks L.J., Olsen M.N., Bacevice A.M., Beebe A., Konstantinopoulou S., Taylor H.G. (2014). Relationship between Sleep, Sleep Apnea, and Neuropsychological Function in Children with Down Syndrome. Sleep Breath..

[74] Joyce A., Dimitriou D. (2017). Sleep-Disordered Breathing and Cognitive Functioning in Preschool Children with and without Down Syndrome. J. Intellect. Disabil. Res..

[75] Canessa N., Castronovo V., Cappa S.F., Aloia M.S., Marelli S., Falini A., Alemanno F., Ferini-Strambi L. (2011). Obstructive Sleep Apnea: Brain Structural Changes and Neurocognitive Function before and after Treatment. Am. J. Respir. Crit. Care Med..

[76] Benejam B., Videla L., Vilaplana E., Barroeta I., Carmona-Iragui M., Altuna-Azkargorta M., Valldeneu S., Fernandez S., Giménez S., Iulita F. (2020). Diagnosis of Prodromal and Alzheimer’s Disease Dementia in Adults with Down Syndrome Using Neuropsychological Tests. Alzheimer’s Dement. Diagn. Assess. Dis. Monit..

[77] Mullins A.E., Kam K., Parekh A., Bubu O., Osorio R.S., Varga A.W. (2020). Obstructive Sleep Apnea and Its Treatment in Aging: Effects on Alzheimer’s disease Biomarkers, Cognition, Brain Structure and Neurophysiology. Neurobiol. Dis..

[78] Sharma R.A., Varga A.W., Bubu O., Pirraglia E., Kam K., Parekh A., Wohlleber M., Miller M.D., Andrade A., Lewis C. (2018). Obstructive Sleep Apnea Severity Affects Amyloid Burden in Cognitively Normal Elderly. A Longitudinal Study. Am. J. Respir. Crit. Care Med..

[79] Fernandez F., Edgin J.O. (2013). Poor Sleep as a Precursor to Cognitive Decline in Down Syndrome: A Hypothesis. J. Alzheimer’s Dis. Park..

[80] Fitzpatrick V., Rivelli A., Bria K., Chicoine B. (2020). Heart Disease in Adults with Down Syndrome Between 1996 and 2016. J. Am. Board Fam. Med..

[81] O’Driscoll D.M., Horne R.S., Davey M.J., Hope S.A., Anderson V., Trinder J., Walker A.M., Nixon G.M. (2012). Cardiac and Sympathetic Activation are Reduced in Children with Down Syndrome and Sleep Disordered Breathing. Sleep.

[82] Konstantinopoulou S., Tapia I.E., Kim J.Y., Xanthopoulos M.S., Radcliffe J., Cohen M.S., Hanna B.D., Pipan M., Cielo C., Thomas A.J. (2016). Relationship between Obstructive Sleep Apnea Cardiac Complications and Sleepiness in Children with Down Syndrome. Sleep Med..

[83] Hendrix J.A., Amon A., Abbeduto L., Agiovlasitis S., Alsaied T., Anderson H.A., Bain L.J., Baumer N., Bhattacharyya A., Bogunovic D. (2021). Opportunities, Barriers, and Recommendations in Down Syndrome Research. Transl. Sci. Rare Dis..

[84] Altuna M., Giménez S., Fortea J. (2021). Epilepsy in Down Syndrome: A Highly Prevalent Comorbidity. J. Clin. Med..

[85] Giannasi L., Cruz M.M.e., Rezende T., Dutra M., Nacif S., Oliveira E., Rode S., Nazário L., Silvestre P., Bacigalupo E. (2020). 0804 Sleep Bruxism, Awake Bruxism and Sleep Related Breathing Disorders in Adults with Down Syndrome. Sleep.

[86] Rosen D., Berbert L., Weller E. (2020). High Prevalence of Periodic Limb Movements of Sleep in Children with Down Syndrome. J. Clin. Sleep Med..

[87] Dye T.J., Jain S.V., Simakajornboon N. (2017). Outcomes of Long-Term Iron Supplementation in Pediatric Restless Legs Syndrome/Periodic Limb Movement Disorder (RLS/PLMD). Sleep Med..

